# A quorum sensing antagonist with dual bacterium- and host-directed anti-pathogenic activity against *Pseudomonas aeruginosa*

**DOI:** 10.1080/21505594.2026.2712735

**Published:** 2026-08-14

**Authors:** Dan-Dan Li, Huiyan Li, Wen-Xin Niu, Huayue Li, Joon-Hee Lee

**Affiliations:** a College of Pharmacy, Pusan National University, Busan, Republic of Korea; b School of Chinese Materia Medica, Tianjin University of Traditional Chinese Medicine, Tianjin, People’s Republic of China; c Key Laboratory of Marine Drugs, Ministry of Education, School of Medicine and Pharmacy, Ocean University of China, Qingdao, China; d Research Institute for Drug Development, Pusan National University, Busan, South Korea

**Keywords:** *Pseudomonas aeruginosa*, anti-pathogenic therapy, quorum sensing inhibition, anti-biofilm, (R)-6’-Debromohamacanthin B, host immune modulation

## Abstract

*Pseudomonas aeruginosa* is a major opportunistic pathogen whose clinical management is increasingly compromised by antibiotic resistance and biofilm-associated persistence. Anti-pathogenic strategies that attenuate virulence without inhibiting bacterial growth offer a promising alternative to conventional antibiotics. Here, we identify the *Spongosorites* sp-derived bisindole alkaloid (R)-6’-debromohamacanthin B (RDB) as a potent dual bacterium- and host-directed anti-pathogenic agent against *P. aeruginosa*. RDB suppressed quorum sensing (QS) activity and markedly inhibited biofilm formation at growth-independent concentrations starting at 100 pM. Mechanistically, RDB functionally interfered with several central QS regulators, including LasR, RhlR, PqsR, and QscR, consistent with network-level attenuation of QS signaling, and reduced intracellular c-di-GMP levels associated with biofilm regulation. Beyond its bacterium-directed activity, RDB enhanced macrophage-mediated phagocytosis and intracellular bacterial clearance while limiting excessive production of pro-inflammatory mediators. In a murine wound infection model, RDB significantly reduced infection severity, restricted systemic dissemination, and promoted wound healing without detectable toxicity. Collectively, these findings demonstrate that RDB exerts dual bacterium-directed and host-directed activities, simultaneously attenuating virulence and biofilm formation while reinforcing host defense and controlling pathological inflammation. This multifaceted mode of action positions RDB as a promising anti-pathogenic therapeutic candidate for the management of *P. aeruginosa* infections without promoting antimicrobial resistance.

## Graphical abstract

**Figure:**
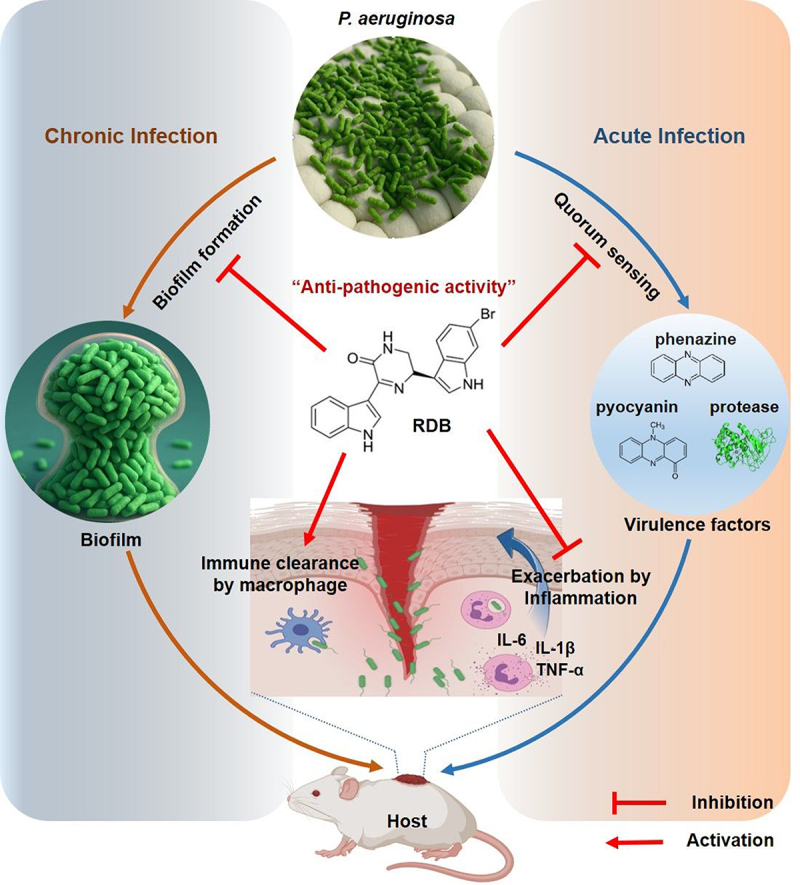

## Introduction

The global escalation of antibiotic resistance has profoundly limited the effectiveness of conventional antimicrobial therapies [1]. This challenge is particularly acute for opportunistic pathogens such as *Pseudomonas aeruginosa*, which exhibits intrinsic resistance, rapidly acquires additional resistance determinants, and forms highly tolerant biofilms [2]. Biofilm-associated infections are especially difficult to eradicate, frequently resulting in chronic or recurrent disease despite aggressive antibiotic treatment [3,4]. These limitations underscore the urgent need for therapeutic strategies that move beyond bactericidal or bacteriostatic paradigms.

Anti-pathogenic approaches have emerged as a promising alternative to traditional antibiotics. Rather than targeting bacterial viability, anti-pathogenic agents attenuate disease by interfering with virulence mechanisms or by modulating host responses [3,4]. Because they do not directly inhibit growth, such agents are expected to impose reduced selective pressure for resistance development [3,4]. Within this framework, two complementary strategies are increasingly recognized: bacterium-directed anti-virulence compounds that suppress pathogenic determinants, and host-directed modulators that enhance protective immunity or restrain excessive inflammation [3,5–7].

*P. aeruginosa* represents a compelling target for anti-pathogenic intervention. Its pathogenicity is orchestrated by interconnected quorum sensing (QS) networks – including the Las, Rhl, Pqs, and QscR systems – which coordinately regulate virulence factor production, biofilm maturation, and adaptive responses [7,8]. Disruption of QS signaling, particularly at the level of receptor activation, has therefore been widely pursued as an antivirulence strategy [5]. However, many reported QS inhibitors target a single receptor or pathway, exhibit modest potency, or lack *in vivo* validation [5]. Given the hierarchical and partially redundant architecture of QS circuitry in *P. aeruginosa*, effective attenuation of virulence may require simultaneous interference with multiple regulatory nodes.

At the molecular level, *P. aeruginosa* produces two principal classes of QS signal molecules: acyl homoserine lactones (acyl-HSLs), including N-3-oxododecanoyl-HSL (3OC12-HSL) and N-butyryl-L-HSL (C4-HSL), and 2-alkyl-4-quinolones (AQs), including 3,4-dihydroxy-2-heptylquinoline (PQS) and 4-hydroxy-2-heptylquinoline (HHQ) [6–8]. These autoinducers bind to their cognate transcriptional regulators – LasR and QscR for 3OC12-HSL, RhlR for C4-HSL, and PqsR for AQs – thereby activating the expression of genes associated with biofilm formation and virulence factor production [9–11]. Accordingly, QS inhibitors that interfere with signal – receptor binding or receptor activation have been proposed as promising therapeutic agents for controlling infections caused by pathogenic bacteria, including *P. aeruginosa* [12].

In parallel, host immune responses critically shape infection outcomes. While macrophage-mediated bacterial clearance is essential for host defense, excessive inflammatory activation contributes to tissue damage and worsened clinical manifestations. Notably, *P. aeruginosa* further exacerbates infection by producing lipopolysaccharide (LPS), exotoxin A, and extracellular polymeric substances (EPS; major biofilm matrix components), which robustly stimulate macrophage activation and inflammatory signaling [13]. Therefore, a compound capable of simultaneously inhibiting QS-driven virulence and biofilm formation while attenuating excessive host inflammatory responses would represent a particularly attractive anti-pathogenic strategy against *P. aeruginosa* infection.

Marine natural products provide a rich and chemically diverse reservoir for the discovery of novel bioactive molecules [14]. In the present study, we screened structurally diverse compounds isolated from marine invertebrates for anti-QS and anti-biofilm activity [15]. We identified the bisindole alkaloid (R)-6’-debromohamacanthin B (RDB) as a potent anti-pathogenic agent against *P. aeruginosa*. Here, we demonstrate that RDB simultaneously suppresses multiple QS regulators, attenuates QS-controlled virulence phenotypes, disrupts biofilm development through modulation of intracellular signaling, and exerts these effects at growth-independent, low nanomolar concentrations. Importantly, RDB also enhances macrophage-mediated bacterial clearance while limiting excessive inflammatory mediator production. Using a murine wound infection model, we further show that RDB reduces infection severity and promotes tissue repair *in vivo*. Collectively, our findings establish RDB as a multifunctional anti-pathogenic compound that integrates multi-node QS inhibition with host-directed immunomodulation, highlighting its potential as a lead scaffold for new therapeutics against *P. aeruginosa*.

## Materials and methods

### Bacterial strains, plasmids, and culture conditions

Bacterial strains and plasmids used in this study are listed in Table S1. Strains were cultured in various media depending on the experiment or bacterial species (Table S2), including Luria-Bertani (LB; 10 g/L tryptone, 5 g/L yeast extract, 5 g/L NaCl), 1/4-strength LB with 0.1% mannitol, M63-Gly (3 g/L KH_2_PO_4_, 7 g/L K_2_HPO_4_, 2 g/L (NH_4_)_2_SO_4_, 1 mM MgSO_4_, 0.2% glycerol), Tryptic Soy Broth (TSB) with 0.5% glucose, LB with 2% glucose, or Brain Heart Infusion (BHI; 8 g/L brain heart infusion solids, 5 g/L peptic digest of animal tissue, 16 g/L pancreatic digest of casein, 5 g/L NaCl, 2 g/L glucose, 2.5 g/L disodium hydrogen phosphate). Protein expression from pJN105-based plasmids was induced with 0.4% L-arabinose. Antibiotics were used at the following concentrations: carbenicillin (150 µg/mL), ampicillin (100 µg/mL), and gentamicin (10 µg/mL).

### Biological and technical replicates

All *in vitro* experiments were independently repeated at least three times using biological replicates unless otherwise stated. For reporter assays, biofilm assays, and cytokine measurements, each experiment included at least three technical replicates per condition. *In vivo* experiments were performed using randomly assigned mice, and group sizes are indicated in the corresponding figure legends. No data points were excluded from analysis.

### QS reporter assays

QS inhibition was evaluated using *P. aeruginosa* reporter strains harboring QS-responsive promoter – reporter constructs, as previously described [7]. The plasmids used are listed in Table S1. For *E. coli* reporter assays, DH5α was transformed with pJN105L (LasR expression plasmid) + pSC11 (*lasI*_p_-*lacZ* fusion plasmid reflecting LasR activity) or pJN105Q (QscR expression plasmid) + pJL101 (PA1897_p_-*lacZ* fusion plasmid reflecting QscR activity) or pJN105P (PqsR expression plasmid) + pJL301(*pqsA*_p_-*lacZ* fusion plasmid reflecting PqsR activity) and cultured in LB with gentamicin (10 µg/mL) and ampicillin (100 µg/mL) at 37°C. At OD_600_ = 0.3, 3OC12-HSL (50 nM for LasR; 100 nM for QscR), PQS (50 nM for PqsR activity), 0.4% arabinose, and various concentrations of RDB were added, and β-galactosidase activity was measured after 90 min. For *P. aeruginosa* reporter assays, PAO1 carrying pSC11, pJL101, pJL501 (*rhlA*p-*lacZ* fusion plasmid reflecting RhlR activity), or pJL301 (*pqsA*p-*lacZ* fusion plasmid reflecting PqsR activity) was cultured in LB with carbenicillin (150 µg/mL) at OD_600_ = 0.02 for 5 h with or without RDB, and QS activity was determined by β-galactosidase assay. The QS activity was normalized to bacterial cell density (OD_600_) to exclude effects attributable to slight difference in growth. RDB concentrations were selected based on preliminary growth curve analyses confirming no impact on bacterial viability under the conditions tested.

### β-galactosidase activity assay

β-galactosidase activity was measured using the Galacto-Light Plus kit (Applied Biosystems, USA, T1011) as previously described [7]. Briefly, cultures treated with or without RDB were measured at 600 nm using a GloMax®-Multi Microplate Multimode Reader (Promega, USA). An equal volume (100 μL) of culture was mixed with 10 μL chloroform, vortexed vigorously, and incubated at room temperature for 15 min. After centrifugation, 10 μL of the supernatant was transferred to a white 96-well plate, mixed with 100 μL substrate solution, and incubated for 1 h in the dark at room temperature. The reaction was stopped by adding 150 μL Accelerator II buffer, and luminescence was measured using the same reader. β-galactosidase activity was expressed as luminescence/OD_600_.

### Total protease activity

Total protease activity assay was performed as previously described [9], with slight modifications. PAO1 was grown overnight in LB broth and then inoculated into fresh medium with or without the compounds, followed by incubation at 37°C with shaking at 180 rpm. At OD_600_ = 0.2, cells were pelleted at 4°C and culture supernatants (CSs) were collected and filtered (0.2 μm). Five microliters of CSs were spotted onto skim milk agar plates (0.5% skim milk, 0.5% peptone, 0.1% glucose, 1.5% agar) and incubated overnight at 37°C. Protease activity was determined by measuring clear zone diameters.

### Virulence assay of P. aeruginosa

Virulence assay was performed as previously described [9,16]. Filtered CSs were concentrated 10× using 10 kDa cutoff Centricon (Vivaspin; Sartorius, 1610011VS). Five microliters of concentrated CSs were injected into *Tenebrio molitor* larvae, which were maintained at 25°C for 3 days. Larval survival was then recorded.

### Biofilm analyses

Biofilm analyses were performed as previously described [17,18]. For static biofilm assays, overnight cultures of *P. aeruginosa*, *Escherichia coli*, *Bacillus subtilis*, *Staphylococcus aureus*, *Salmonella enterica*, and MDR strains (Table S1) were diluted 1:100 in the indicated media with or without test compounds at concentrations of 100 pM, 1 nM, 10 nM, or 100 nM. Then, 200 μL of each diluted culture was seeded into 96-well plates. After 24 h incubation at 37°C without shaking, OD_600_ was measured, plates were washed with water, dried, and stained with 180 μL of 0.1% crystal violet for 10 min. After staining, the crystal violet solution was discarded, and the 96-well plates were gently washed with water to remove unbound dye and then air-dried. The bound dye was solubilized with 200 μL of 30% acetic acid and quantified at 600 nm using a GloMax®-Multi Reader. For flow-cell assays, GFP-expressing *P. aeruginosa* (PAO1-GFP, Table S1) was diluted to OD_600_ = 0.5, introduced into flow chambers, and incubated for 2 h at room temperature. Chambers were perfused with 1% TSB containing 1 mM IPTG, with or without 10 nM RDB, at 0.25 mL/min for 4 days. Biofilms were visualized by fluorescence (Zeiss Axioskop FL) and confocal microscopy (ZEISS LSM 800 or Olympus FV10i). For the quantitative evaluation of flow-cell biofilms, the total fluorescence intensity of the final mature biofilms was quantified using ImageJ software (NIH, Bethesda, MD, USA) and expressed in arbitrary units (a.u.).

### EPS expression analysis

Flow-cell biofilms were stained with Texas Red – conjugated Concanavalin A (100 μM) for 30 min, followed by washing with flow medium (1% TSB) for 30 min. EPS was visualized using a ZEISS LSM 800 confocal microscope (excitation 495 nm, emission 615 nm), and 3D biofilm images were reconstructed and analyzed with Imaris 9.5.1.

### Intracellular c-di-GMP measurement

Intracellular c-di-GMP levels were determined using a *cdrA*_p_-*lacZ* reporter plasmid (pSKcdrA, Table S2), as previously described [7]. The reporter strain (PAO1 harboring pSKcdrA) was grown in LB broth containing 150 μg/ml carbenicillin. Overnight cultures were diluted 1:100 into fresh LB broth, treated with 10 μM compounds for 5 h, and analyzed by β-galactosidase assay.

### Molecular docking analysis

Crystal structures of LasR (4NG2), PqsR (6Q7U), QscR (3SZT), and RhlR (8B4A) were obtained from the Protein Data Bank (https://www.rcsb.org). Protein preparation involved the removal of crystallographic water molecules, addition of hydrogen atoms at pH 7.4 using the CHARMm force field, assignment of protonation states, and a short energy minimization to relieve steric clashes. Ligand preparation included conformation generation and energy minimization. The binding grid was generated around the original co-crystallized ligand with a radius of approximately 10 Å. Docking was carried out using the LibDock protocol, with parameters set as follows: maximum conformations = 255, and docking tolerance = 0.25.

### Molecular dynamics simulation analysis

Molecular dynamics (MD) simulations for RDB in complex with each of the four target proteins (LasR, PqsR, QscR, and RhlR) using the GROMACS. The simulation procedure included the following steps: assignment of force field parameters (CHARM36m), consideration of solvent effects using an explicit water box, and energy minimization to relax the initial structure. Subsequently, dynamic simulations were carried out through a three-step process including heating, equilibration, and production sampling. Production MD runs were performed for 100 ns. Post-simulation analyses, including root-mean-square deviation (RMSD), root-mean-square fluctuation (RMSF), and intermolecular hydrogen bond occupancy, were conducted to assess the conformational stability of the protein-ligand complexes and the residue-level flexibility of the protein.

### Cell culture, drug treatment, and cell viability assay

RAW 264.7 macrophages (ATCC, Rockville, MD, USA) were cultured at 37°C in a humidified 5% CO_2_ incubator using DMEM/high glucose medium (HyClone, Logan, UT, USA) supplemented with 10% heat-inactivated fetal bovine serum (Gibco, Grand Island, NE, USA), 100 U/mL penicillin, and 100 μg/mL streptomycin (HyClone). Cells were seeded into 96-well plates (1 × 10^4^ cells/well) or 6-well plates (1 × 10^5^ cells/well), pretreated with RDB for 1 h, and subsequently stimulated with lipopolysaccharide (LPS, 100 ng/mL) or PAO1 (MOI = 100). For cell viability assays, cells were treated with RDB for 24 h, followed by the addition of 20 μL MTT solution (5 mg/mL) to each well and incubation for 3 h in the dark. After removing the supernatant, 150 μL DMSO was added to solubilize the formazan crystals, and absorbance was measured at 490 nm using a microplate reader.

### Phagocytosis and clearance of P. aeruginosa

RAW 264.7 cells were infected with PAO1-GFP (MOI = 100) for 1 h and then treated with gentamicin (100 μg/mL) to eliminate extracellular bacteria. After washing with PBS, intracellular fluorescence was quantified as relative fluorescence units (RFU) using a GloMax®-Multi Microplate Multimode Reader (excitation 485 ± 20 nm, emission 528 ± 20 nm). Intracellular PAO1-GFP was further examined by flow cytometry (FL1-H, 530/30 nm bandpass filter) and confocal microscopy (ZEISS LSM 800). The flow cytometry data and FL1-H histogram were analyzed with FlowJo software. For clearance assays, infected cells were incubated in fresh DMEM/high glucose medium containing gentamicin (100 μg/mL) for 5 h to kill remaining extracellular bacteria, and bacterial clearance was quantified as RFU using the GloMax® reader.

### Measurement of inflammatory factor production

Nitric oxide (NO) production was induced by LPS stimulation. After drug and LPS treatment, 80 μL of culture supernatant was mixed with 80 μL of Griess reagent in a 96-well plate and incubated for 20 min at 37°C in the dark. Absorbance was measured at 520 nm using a microplate reader, with sodium nitrite as the standard and dexamethasone (10 μM) as the positive control. For cytokine assays, RAW 264.7 cells were treated with RDB (1, 5, 10, and 20 μM) and LPS for 24 h, and the levels of TNF-α, IL-1β, and IL-6 were determined using ELISA kits (BioLegend, catalog No. 430904 for TNF-α, 433404 for IL-1β, 431304 for IL-6). For COX-2 and iNOS analysis, cells were treated with RDB and infected with PAO1, then lysed in lysis buffer. Protein concentrations were measured using a BCA kit, and equal amounts of protein were separated by SDS-PAGE, transferred to PVDF membranes, blocked with 5% milk, and incubated overnight at 4°C with primary antibodies (anti-COX-2 and anti-iNOS). After incubation with secondary antibodies for 1 h, protein bands were visualized with a ChemiDoc™ Touch Imaging System (Bio-Rad), and band intensities were quantified using ImageJ software.

### Mouse infection experiments

Mouse infection experiments were performed as previous described [19]. All animal experiments were conducted in accordance with institutional ethical guidelines. Mice were divided into four groups: control, RDB, PAO1, and RDB + PAO1. For the control group, PBS, used as the vehicle for RDB, was applied directly to the wound site. Mice were monitored daily for clinical signs, body weight changes, and wound progression throughout the infection experiments, and at the indicated time points, tissues were harvested for bacterial burden quantification, and histopathological analysis. Seven-week-old male Jcl:ICR mice (Samtaco BIO, Osan, Korea) were anesthetized with 1.2% avertin, and the dorsal hair was shaved using an electric razor. Full-thickness excisional skin wounds (6 mm punch) were created and subsequently infected with *P. aeruginosa* PAO1 (4.2 × 10^6^ CFU per 28.27 mm^2^), with or without RDB (5.43 μg/kg, a dose targeting approximately 10 μM to simultaneously promote anti-virulence and immunomodulatory effects). For the treatment group, RDB was topically applied to completely cover the wound area immediately after infection (day 0) and re-administered as a second dose on day 2 post-infection. Each group of mice was housed in separate cages. Wound images were captured every two days to monitor wound progression. On day 6 post-infection, mice were euthanized using CO_2_, and wound tissues were collected for further analyses. Wound areas were quantified using ImageJ software. Mice were randomly assigned to each experimental group; the PAO1 + RDB group consisted of six mice, while the remaining groups contained five mice each. The sample size of five mice per group was determined based on an a priori power analysis performed using G*Power 3. The experimental unit was a single mouse. All mice underwent organ index assessment, blood analysis, and hematoxylin and eosin (H&E) staining. The order of procedures and measurements was kept consistent across all groups to minimize variability. All experimenters were aware of group assignments throughout the study.

### Bacterial detection in blood, histological analysis, and organ index

On day 6 in the mouse infection experiments, tail blood was taken, diluted with PBS, and spread on VBMM agar plates (0.3% trisodium citrate, 0.2% citric acid, 1% K_2_HPO_4_, 0.35% NaNH_4_PO_4_, 1 mM MgSO_4_, 100 mM CaCl_2_, pH 7.0, 1.5% agar) to count *P. aeruginosa*. Plates were incubated overnight at 37°C, and colony numbers were counted. For histological examination, cross-sectional full-thickness skin specimens were collected on day 6, embedded in OCT compound (Tissue-Tek, USA), frozen on dry ice, sectioned at 5 μm using a cryostat microtome (CM1860, Leica, Germany), and stained with hematoxylin and eosin (H&E). Histopathological changes were observed and imaged using a Zeiss Axioskop FL microscope. To calculate organ indexes, the heart, liver, spleen, lungs, and kidneys were dissected and weighed, and each organ index was calculated as organ weight (mg) divided by body weight (g).

### Ethical statement

All animal experiments were performed in compliance with the ARRIVE guideline and in accordance with the Animals (Scientific Procedures) Act 1986, its associated guidance, and EU Directive 2010/63 on the protection of animals used for scientific purposes. This study involved no human participants. All experimental protocols involving mice were reviewed and approved by the Experimental Animal Welfare Ethics Committee of Pusan National University (PNU-2024–0360).

### Statistical analysis

All quantitative data are presented as mean ± SEM. Statistical analyses were performed using GraphPad Prism 5. For comparisons among multiple groups, one-way ANOVA followed by Tukey’s post hoc test was applied. For experiments involving comparison between two groups only (Figure 1 and 6(G)), statistical significance was determined using an unpaired two-tailed Student’s t-test. A *p* value < 0.05 was considered statistically significant.

**Figure 1.**
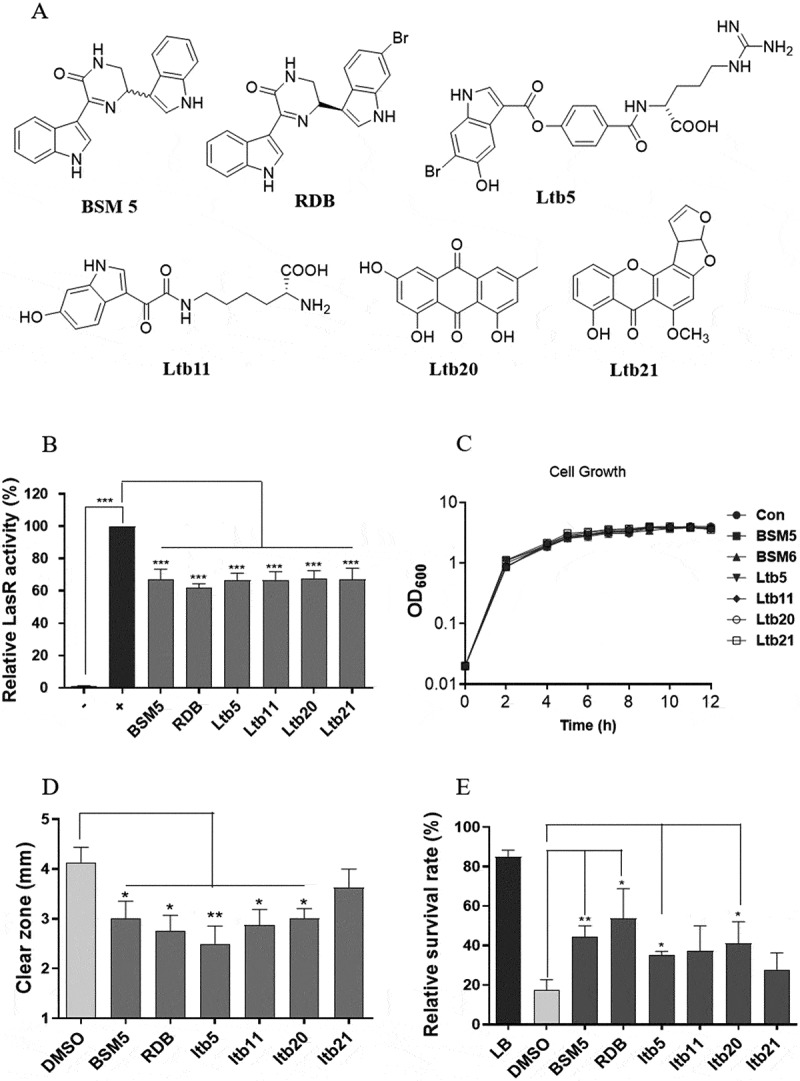
Six marine-derived compounds with QS–inhibitory and anti-virulence activity. (A) Molecular structures of the six marine natural compounds initially identified through their ability to inhibit LasR activity. (B) Anti-LasR activity of the six compounds was assessed using an *E. coli* dual-plasmid reporter system. (Data are presented as mean ± SEM. *n* = 3 for each group.) (C) Growth curves of *P. aeruginosa* treated with the six compounds. (Data are presented as mean ± SEM. *n* = 2 for each group.) (D) Total extracellular protease activity was evaluated using a skim milk agar assay, and the diameters of the clear zones were measured. (Data are presented as mean ± SEM. *n* = 4 for each group.) (E) Virulence was assessed in *Tenebrio molitor* larvae, and survival rates were analyzed. (Data are presented as mean ± SEM. *n* = 4 for each group.) *, *р* < 0.05; **, *р* < 0.01; ***, *р* < 0.001.

## Results

### Identification of marine-derived compounds with QS – inhibitory and anti-virulence activity

To identify marine natural products with anti-QS activity, thirty compounds isolated from the marine sponge *Spongosorites* sp., the ascidian *Herdmania momus*, and the brittle star *Ophioplocus japonicus* (Figure S1) [20–24] were screened using a β-galactosidase reporter assay [7]. Six compounds (BSM5, RDB, Ltb5, Ltb11, Ltb20, and Ltb21, see Figure 1(A)) significantly inhibited LasR-dependent transcription (Figure 1(B)) without affecting the growth of *P. aeruginosa* (Figure 1(C)), indicating QS inhibition independent of bacterial viability.

To assess whether QS inhibition translated into reduced virulence, extracellular protease activity and *Tenebrio molitor* infection assays were performed [9,10]. At 10 μM, five compounds (BSM5, RDB, Ltb5, Ltb11, and Ltb20) significantly reduced protease activity, and four compounds (BSM5, RDB, Ltb5, and Ltb20) significantly attenuated *in vivo* virulence in the *T. molitor* model (Figure 1(D,E)). Among these, BSM5, RDB, and Ltb20 consistently exhibited both QS-inhibitory and anti-virulence activities without affecting bacterial growth and were selected for subsequent biofilm analyses.

### RDB potently inhibits biofilm formation and EPS production

Among the three selected compounds, RDB exhibited the most potent anti-biofilm activity. RDB significantly inhibited biofilm formation in both wild-type and a multidrug-resistant *P. aeruginosa* strain (MDR1) at very low concentrations, ranging from 100 pM to 1 μM for PAO1 and from 100 nM to 1 μM for MDR1, without affecting bacterial growth (Figure 2(A,B)). In contrast, BSM5 showed only weak inhibition, and Ltb20 had negligible effects.

**Figure 2.**
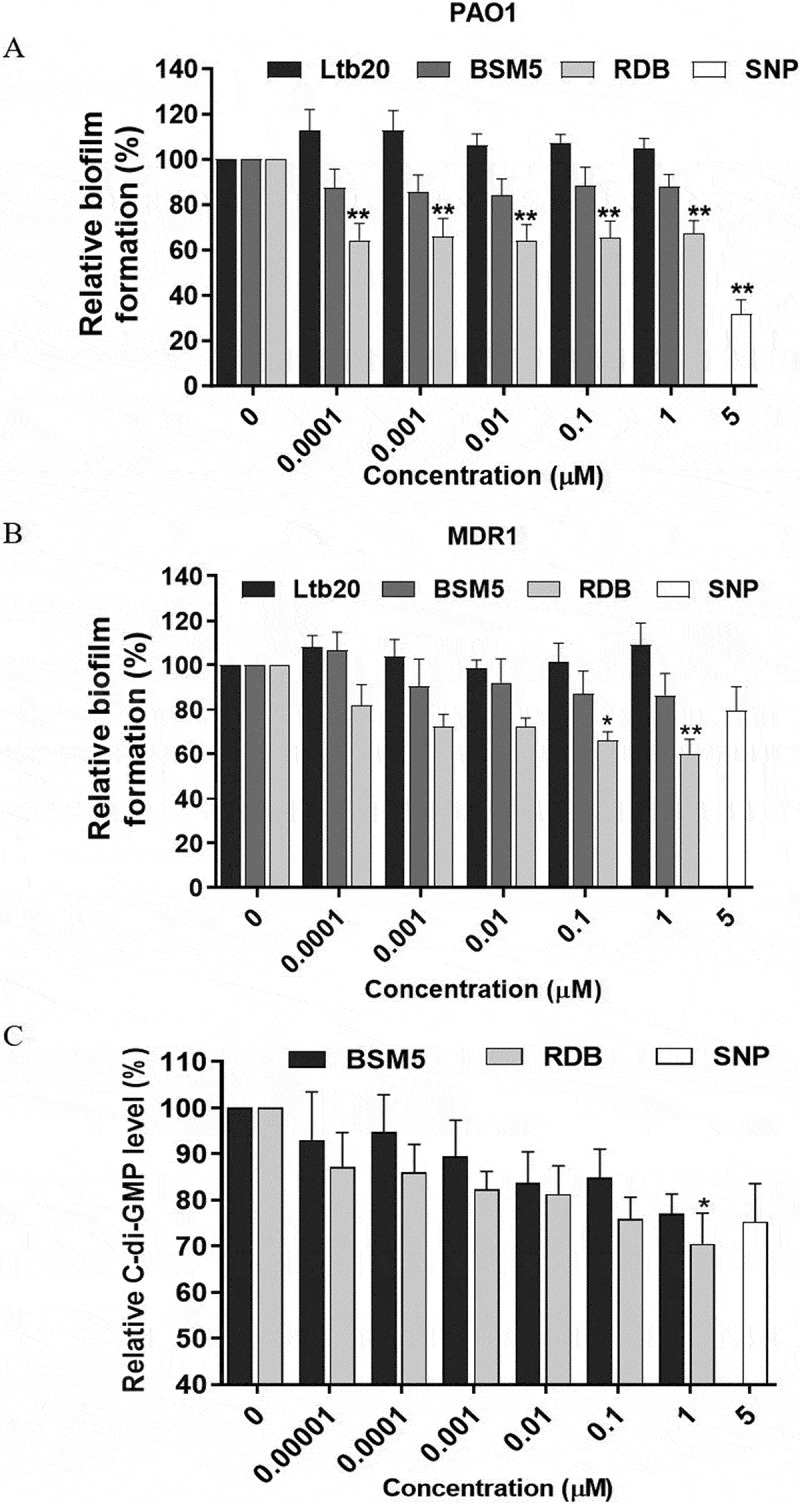
RDB inhibits biofilm formation by reducing intracellular c-di-GMP levels. (A–B) Static biofilm formation assays were performed to evaluate the anti-biofilm activities of RDB, Ltb20, and BSM5. SNP (5 μM) served as a positive control. (Data are presented as mean ± SEM. For Figure 2(A), *n* = 4 for Ltb20 group, *n* = 8 for RDB and BSM5 group, *n* = 11 for SNP group. For Figure 2(B), *n* = 3 for Ltb20 group, *n* = 5 for RDB and BSM5 and SNP group.) (C) Intracellular c-di-GMP levels were quantified by a β-galactosidase assay using *P. aeruginosa* harboring the pSkcdra plasmid. *, *р* < 0.05; **, *р* < 0.01. (Data are presented as mean ± SEM. *n* = 3 for each group.).

Because intracellular cyclic di-GMP (c-di-GMP) is a central messenger molecule to regulate biofilm formation and extracellular matrix production [11], intracellular c-di-GMP levels were monitored using a *cdrAp – lacZ* reporter strain [7]. RDB markedly reduced intracellular c-di-GMP levels at 1 μM, demonstrating greater efficacy than BSM5 (Figure 2(C)). Notably, at 100 nM, RDB inhibited MDR1 biofilm formation more effectively than 5 μM sodium nitroprusside (SNP), a well-established biofilm inhibitor [25] (Figure 2(B)). Based on its potent QS-inhibitory, anti-virulence, and anti-biofilm activities at growth-independent concentrations, RDB was selected for further mechanistic and functional analyses.

### RDB selectively disrupts P. aeruginosa biofilm architecture

To assess the spectrum of RDB activity, its effects on biofilm formation were evaluated in multiple bacterial species, including *Escherichia coli*, *Bacillus subtilis*, *Salmonella enterica*, *Staphylococcus aureus*, and eight additional multidrug-resistant strains (MDR2–9). RDB significantly inhibited biofilm formation in *P. aeruginosa* and *E. coli*, whereas among the resistant strains, only MDR1 was affected (Figures. 2, S2), indicating selective anti-biofilm activity against *P. aeruginosa*.

Flow-cell biofilm analyses further revealed that treatment with 10 nM RDB for four days markedly reduced overall biofilm biomass (Figure 3(A)) and altered biofilm architecture from a characteristic mushroom-like structure to a flatter and less organized configuration (Figure 3(B)). Quantitative analysis of the total fluorescence intensity confirmed that RDB treatment resulted in a 3.6-fold and 10.4-fold reduction in biofilm formation for Figure 3(A,B), respectively, at the final stage. Consistently, treatment with 1 nM RDB significantly reduced extracellular polymeric substance (EPS) production, as visualized by Texas Red – conjugated concanavalin A staining (Figure 3(C)). These results demonstrate that RDB disrupts *P. aeruginosa* biofilm development and architecture at concentrations that do not affect bacterial growth.

**Figure 3.**
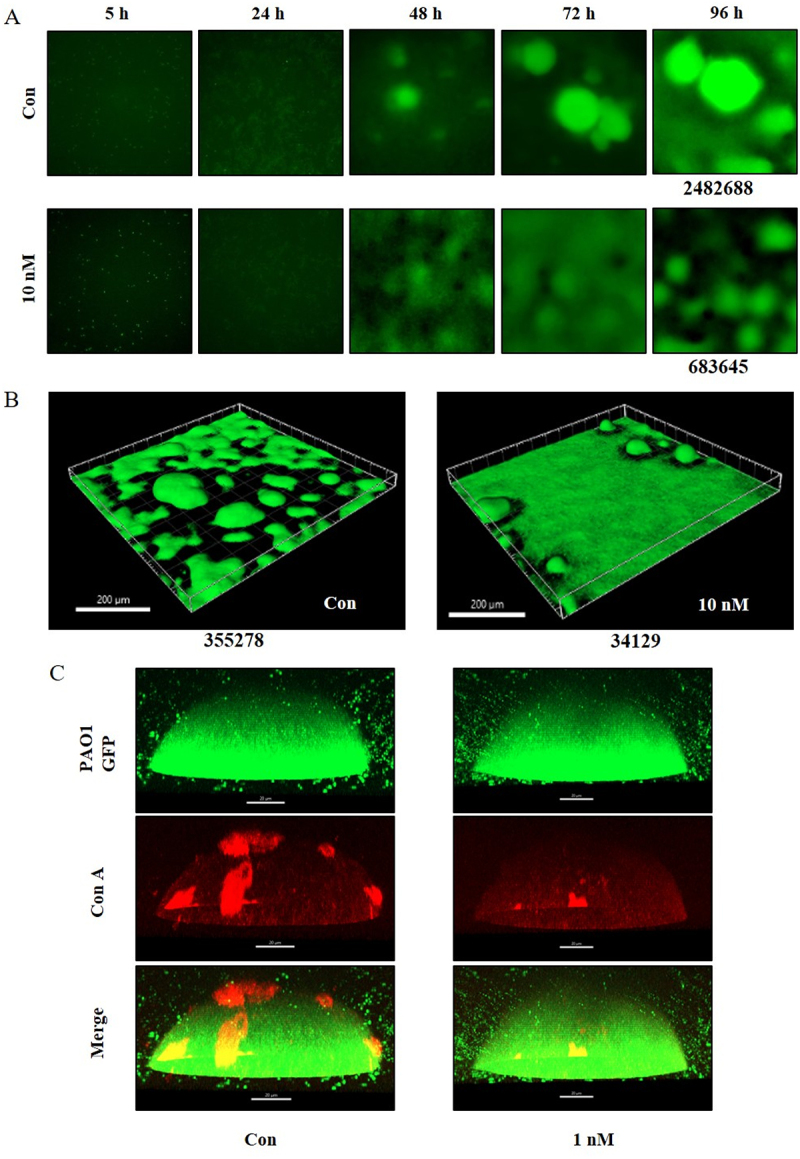
RDB suppresses EPS production and biofilm architecture at low concentrations. (A) PAO1-GFP cells were grown in a flow-cell chamber for 4 days with or without 10 nM RDB, and biofilm development was monitored at 5h, 24h, 48h, 72h and 96h by microscopy. (B) After 96 h, 3D biofilm structures were visualized by confocal microscopy. The quantitative values at the bottom of the panels (at 96 h for A, and the final panels for B) represent the total fluorescence intensity of the mature biofilms quantified using ImageJ, expressed in arbitrary units. (C) PAO1-GFP cells treated with 1 nM RDB for 96 h were stained with Texas Red – conjugated concanavalin a to visualize and quantify EPS.

### RDB suppresses multiple QS regulators at pico- to nanomolar concentrations

To investigate the molecular basis of RDB-mediated QS inhibition, docking simulations were performed with four major QS regulators: LasR, QscR, RhlR, and PqsR. As shown in Table 1, RDB exhibited higher LibDockScores than the corresponding native ligands for all four regulators (3OC12-HSL for LasR and QscR, HHQ for PqsR, and C4-HSL for RhlR) [26–29]. Predicted hydrogen bonding and hydrophobic interactions between RDB and each regulator were comparable to or stronger than those observed for native ligands (Figure 4(A–H); Table S3).

**Figure 4.**
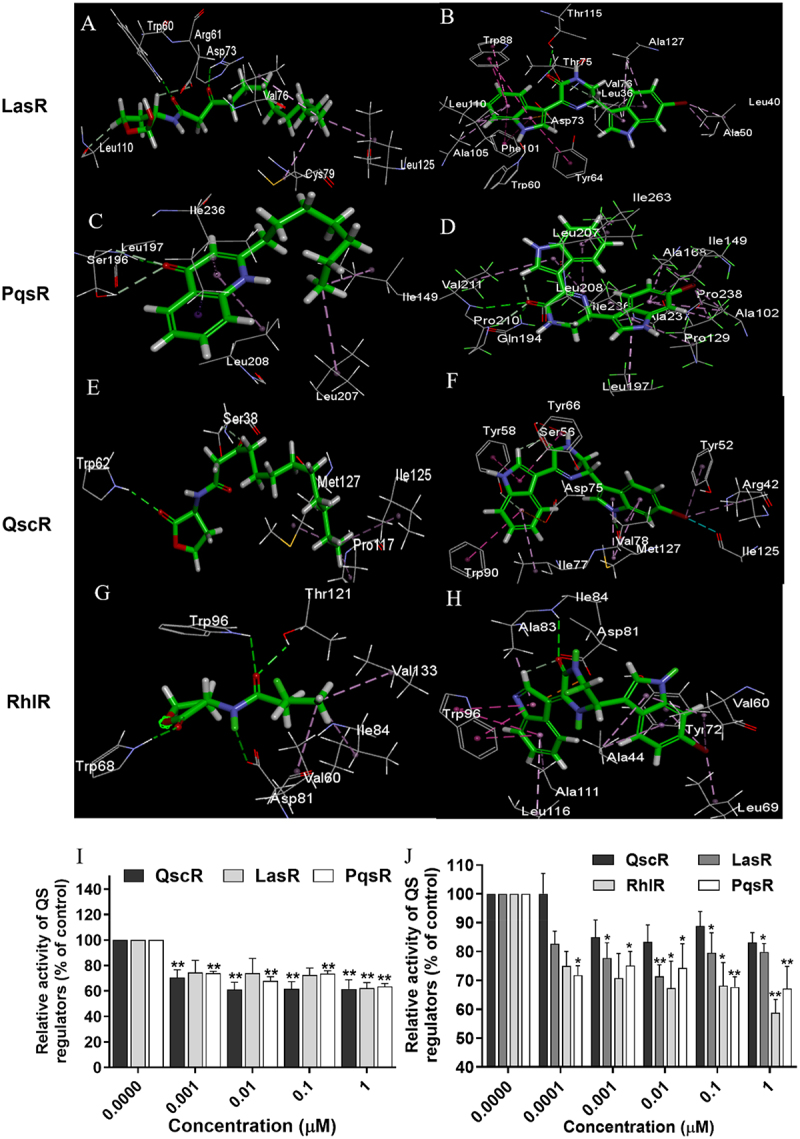
RDB inhibits multiple QS regulators at low concentrations. (A-H) Docking simulations of native ligands (A, C, E, G) or RDB (B, D, F, H) with LasR (A-B), PqsR (C-D), QscR (E-F), and RhlR (G-H). (I) The inhibitory effects of RDB on QS receptors were evaluated using the *E. coli* dual-plasmid reporter system. (Data are presented as mean ± SEM. *n* = 4 for LasR group, *n* = 7 for QscR group, *n* = 9 for PqsR group.) (J) QS inhibition by RDB was further confirmed in a *P. aeruginosa* reporter strain. (data are presented as mean ± SEM. *n* = 7 for LasR and QscR group, *n* = 5 for RhlR and PqsR group.) *, *р* < 0.05; **, *р* < 0.01.

**Table 1.** The LibDockScores of RDB and their original ligands with LasR, PqsR, QscR or RhlR (3OC12-HSL for LasR and QscR, HHQ for PqsR, C4-HSL for RhlR).

LibDockScore	LasR (4NG2)	PqsR (6Q7U)	QscR (3SZT)	RhlR (8B4A)
With original ligand	137.181	93.5114	138.16	81.1341
With RDB	148.074	121.166	151.042	111.272

To further evaluate the binding stability of RDB with four QS target proteins (LasR, PqsR, QscR, and RhlR), molecular dynamics (MD) simulations were performed using GROMACS. As shown in Figure S3A – S3F, RDB formed stable complexes with LasR and PqsR, which exhibited the highest structural stability and the lowest residue fluctuations among the four systems. In contrast, the QscR-RDB and RhlR-RDB complexes displayed comparatively greater conformational flexibility, yet sustained hydrogen-bond interactions were maintained throughout the simulation trajectories (Figure S3G – S3L). Collectively, these results demonstrate that RDB can form relatively stable complexes with all four QS receptors, providing computational evidence for its potential as a multi-target QS inhibitor.

To experimentally validate these findings, plasmids expressing LasR or QscR or PqsR were co-transformed with their cognate *lacZ* reporter plasmids into *E. coli*, and competition assays were performed in the presence of 3OC12-HSL for LasR and QscR, or PQS for PqsR. RDB significantly inhibited QscR- and PqsR-mediated transcriptional activity at concentrations ranging from 1 nM to 1 μM, while a significant inhibition of LasR-mediated activity was observed at 1 μM (Figure 4(I)). Consistent results were obtained in *P. aeruginosa*, where RDB inhibited QS-dependent activities with varying threshold concentrations. Specifically, a statistically significant inhibition was achieved starting from 0.1 nM for PqsR, 1 nM for LasR, and 10 nM for RhlR (Figure 4(J)). Notably, the overall suppressive effect was particularly robust for the RhlR- and PqsR-dependent pathways, whereas RDB-mediated suppression of QscR-dependent activity did not reach statistical significance (Figure 4(J)). These results demonstrate that RDB broadly suppresses multiple QS regulators at very low, growth-independent concentrations.

### RDB enhances macrophage phagocytosis and bacterial clearance

To examine the effects of RDB on host antibacterial defenses, macrophage phagocytic activity was assessed using GFP-labeled *P. aeruginosa*. RDB treatment significantly enhanced macrophage phagocytosis in a dose-dependent manner, as demonstrated by increased intracellular GFP fluorescence (Figure 5(A)), a rightward shift in FL1-H fluorescence by flow cytometry (Figure 5(B)), and fluorescence microscopy (Figure 5(C)).

**Figure 5.**
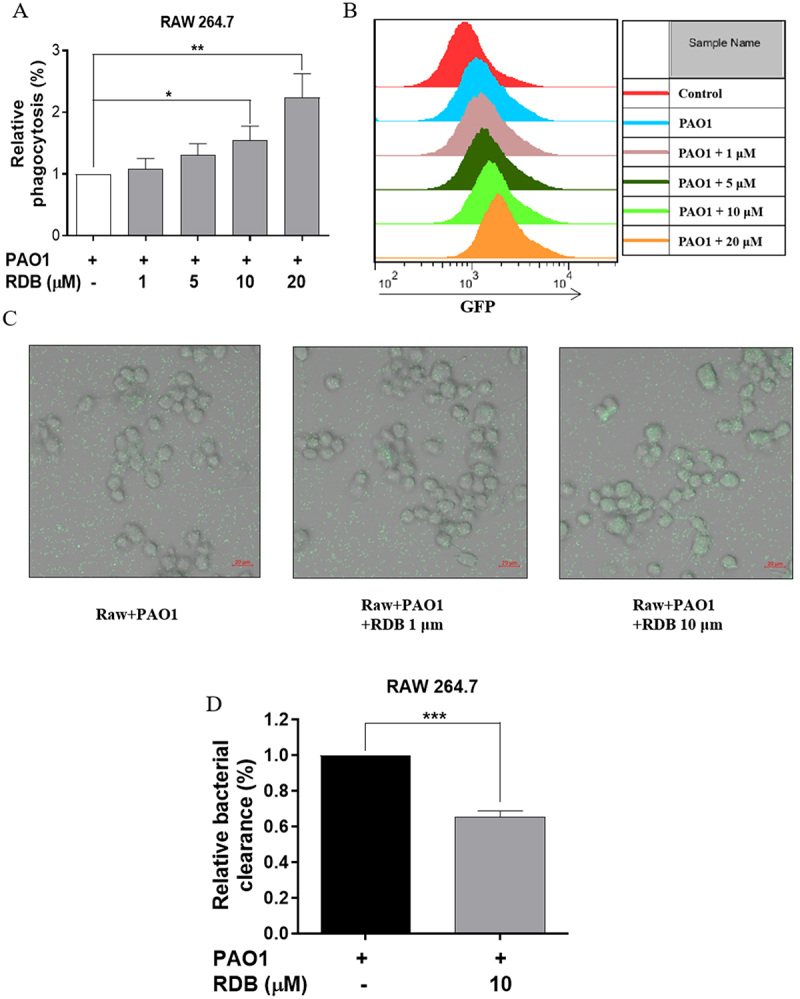
RDB enhances macrophage-mediated phagocytosis and clearance of *P. aeruginosa*. (A-C) RAW 264.7 macrophages were pretreated with RDB and infected with PAO1-GFP. Phagocytosis was quantified by GFP fluorescence (A), flow cytometry (B), and confocal microscopy (C) (Data are presented as mean ± SEM in Figure 5(A). *n* = 6 for each group.). (D) After extracellular bacteria were eliminated with 100 μg/mL gentamicin, RAW 264.7 cells were incubated for 5 h, and surviving bacterial cells within RAW264.7 cells were quantified by fluorescence. (Data are presented as mean ± SEM. *n* = 5 for each group). *, *р* < 0.05; **, *р* < 0.01; ***, *р* < 0.001.

Bacterial clearance was subsequently evaluated by monitoring the reduction of intracellular GFP fluorescence following phagocytosis. RDB treatment significantly accelerated the clearance of internalized bacteria over a 5-hour period (Figure 5(D)). These results indicate that RDB enhances macrophage-mediated uptake and clearance of *P. aeruginosa*.

### RDB suppresses inflammatory mediator production without cytotoxicity

While macrophage-mediated phagocytosis and bacterial clearance are essential for host defense, cytokines produced during the inflammatory response to *P. aeruginosa* infection can sometimes worsen disease by promoting excessive inflammation [13]. To assess whether RDB modulates inflammatory responses, its cytotoxicity was first evaluated in RAW 264.7 macrophages. RDB showed no detectable cytotoxicity at concentrations up to 20 μM in the presence or absence of LPS (Figure 6(A,B)), and concentrations ≤ 20 μM were used for subsequent experiments. RDB pretreatment significantly reduced LPS-induced production of nitric oxide, TNF-α, and IL-1β, as determined by ELISA and immunoblot analyses, whereas IL-6 production was not significantly affected (Figure 6(C–F)). In addition, RDB markedly suppressed LPS-induced expression of COX-2 and iNOS (Figure 6(G)). These findings demonstrate that RDB selectively attenuates inflammatory mediator production while preserving cell viability. Taken together, while these results demonstrate the immunomodulatory potential of RDB, it should be noted that the immunological assays in this study – specifically the phagocytosis and inflammatory mediator evaluations (Figure 5 and 6) – were performed exclusively using the RAW 264.7 cell line. Therefore, further validation in primary macrophage models, such as BMDMs or human primary macrophages, remains necessary to generalize these findings.

**Figure 6.**
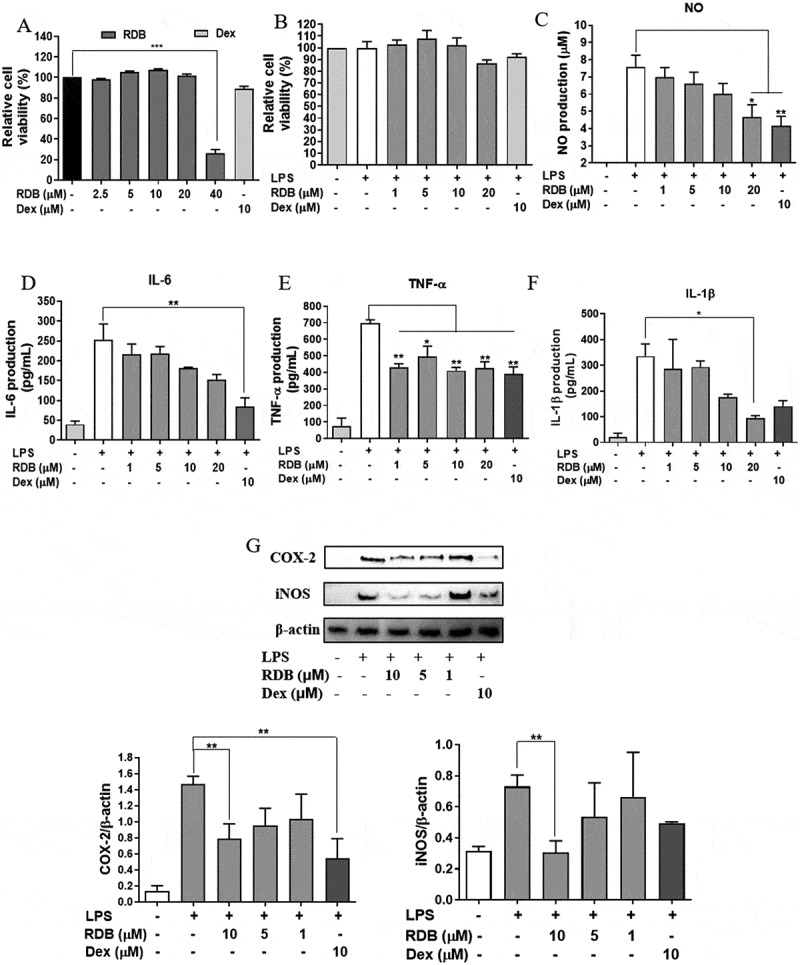
RDB suppresses inflammatory mediator production without cytotoxicity. (A–B) Viability of RAW 264.7 cells treated with RDB was measured with or without LPS stimulation. (Data are presented as mean ± SEM. *n* = 3 for each group.) (C) NO production was measured after RDB treatment under LPS stimulation. (Data are presented as mean ± SEM. *n* = 5 for each group.) (D – F) Levels of IL-6, TNF-α, and IL-1β were quantified by ELISA. (Data are presented as mean ± SEM. *n* = 3 for IL-6 and TNF-α group, *n* = 4 for IL-1β group.) (G) COX-2 and iNOS expression levels were analyzed by western blotting, and band intensities were quantified and are shown below. Dexamethasone (dex) served as a positive control. (Data are presented as mean ± SEM. *n* = 4 for each group.) *, *р* < 0.05; **, *р* < 0.01; ***, *р* < 0.001.

### RDB attenuates P. aeruginosa infection and promotes wound healing in vivo

Based on its growth-independent anti-pathogenic and immunomodulatory activities *in vitro*, the therapeutic potential of RDB was evaluated in a murine wound infection model. Full-thickness wounds were created on the backs of mice and infected with *P. aeruginosa*, resulting in pronounced pus formation and delayed wound closure in untreated mice. In contrast, RDB treatment (5.43 μg/kg) significantly reduced pus formation, accelerated wound closure, and decreased wound size (Figure 7(A,B)). RDB treatment also prevented infection-associated body weight loss, whereas RDB alone had no significant effect in uninfected mice (Figure 7(C)). Systemic bacterial dissemination was markedly reduced in RDB-treated mice, with minimal bacterial detection in the blood on day 6 post-infection (Figure 7(D)). Analysis of organ indices revealed no significant effects on most organs; however, splenic enlargement was observed in infected mice treated with RDB (Figure 7(E,F)).

**Figure 7(a).**
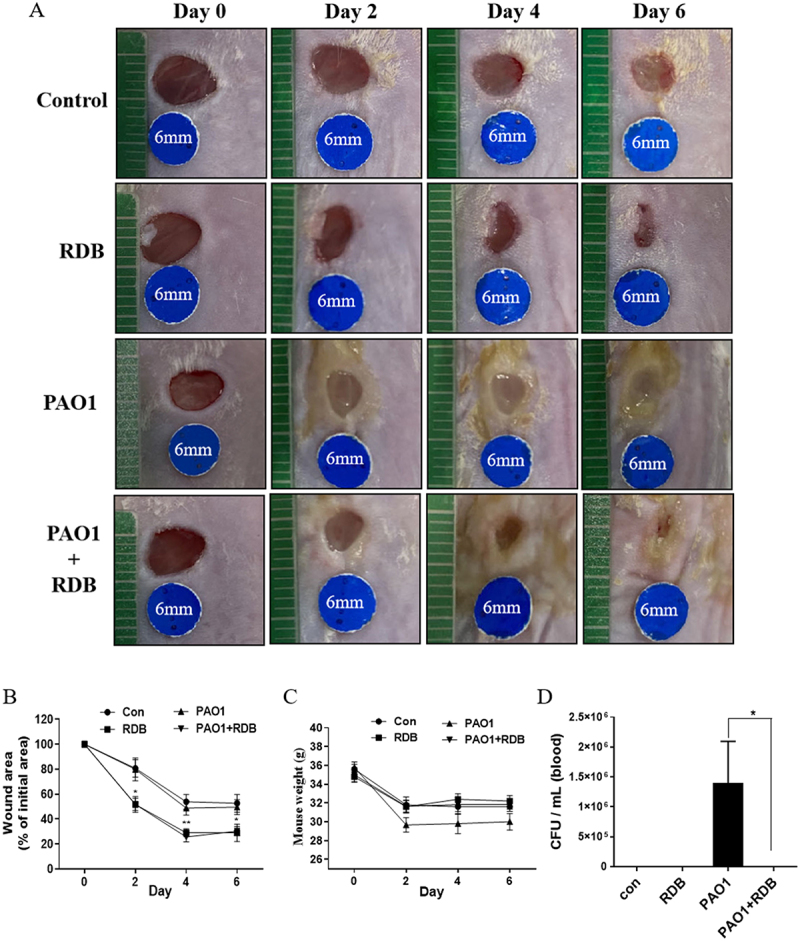
RDB attenuates *P. aeruginosa* infection in a mouse wound model. (A) Mice were infected with PAO1 (4.2 × 10^6^CFU/28.27 mm^2^) on dorsal wounds and treated with RDB (0.1629 μg/28.27 mm^2^; 5.43 μg/kg) for 6 days. Wound progression was monitored. (B) Wound area was quantified using ImageJ from day 0 to day 6. (C) Mouse body weight was recorded during infection and treatment. (D) Bacterial burden in blood was determined by CFU counting. (E) On day 6, mice were sacrificed and organ indices of the heart, liver, spleen, lungs, and kidneys were calculated. (F) The spleens were excised after sacrifice, and their sizes were compared. *, *р* < 0.05; **, *р* < 0.01.

**Figure 7(b).**
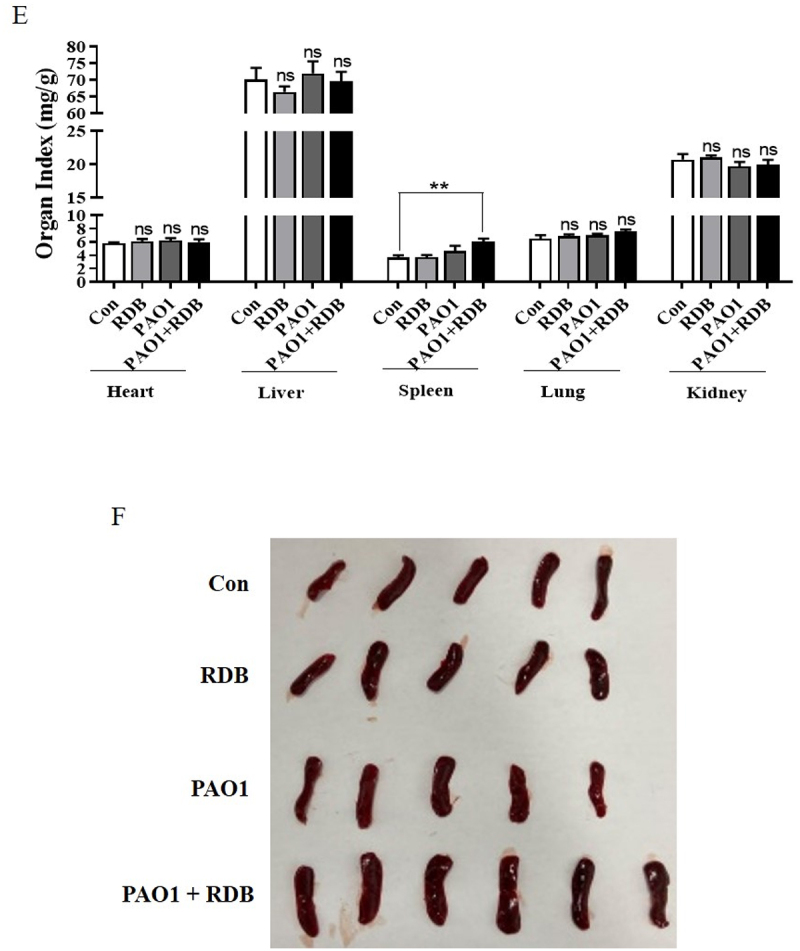
Continued.

Histological examination of infected wounds showed extensive inflammatory cell infiltration in untreated mice, which was markedly reduced by RDB treatment (Figure 8(A)). Consistently, RDB significantly promoted wound closure in a scratch assay using HaCaT keratinocytes (Figure 8(B)). Together, these results demonstrate that RDB mitigates *P. aeruginosa* infection severity and promotes wound healing *in vivo*.

**Figure 8.**
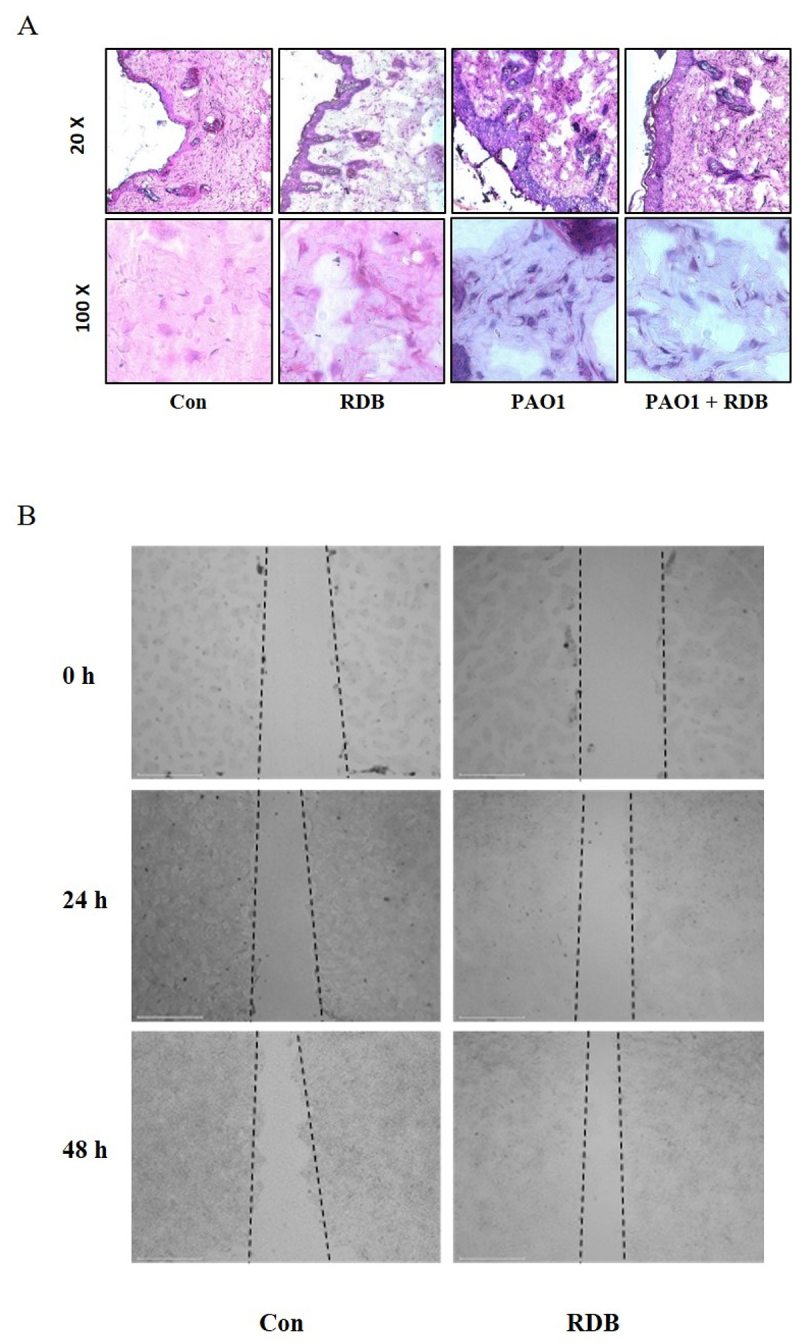
RDB promotes wound healing in infected tissues and in HaCaT cell monolayers. (A) Infected wound tissues were analyzed by H&E staining. (B) A scratch wound-healing assay was performed in HaCaT cells to evaluate the effects of RDB on wound closure.

## Discussion and conclusion

The increasing prevalence of antibiotic resistance in *P. aeruginosa* underscores the need for therapeutic strategies that attenuate pathogenicity without imposing strong selective pressure on bacterial survival. In this study, we identify RDB as a multifunctional anti-pathogenic agent that integrates bacterium-directed antivirulence activity with host-directed immunomodulation. A principal advance of this work is the demonstration that RDB functionally suppresses multiple QS regulators within the interconnected QS network of *P. aeruginosa*. Rather than targeting a single QS pathway, RDB interferes with several central QS nodes, resulting in broad attenuation of QS-controlled phenotypes at pico- to nanomolar, growth-independent concentrations. Given the hierarchical and partially redundant architecture of QS signaling, such multi-node interference is likely to provide more comprehensive and durable suppression of virulence than single-target approaches. These findings position RDB as a network-level QS antagonist rather than a pathway-restricted inhibitor.

Beyond QS regulation, RDB disrupts biofilm development in a manner consistent with modulation of intracellular regulatory circuitry, including c-di-GMP – associated pathways. Because biofilm-associated infections contribute substantially to antibiotic tolerance and chronic persistence [12,14], the ability of RDB to impair biofilm architecture without affecting planktonic growth highlights its therapeutic relevance.

Importantly, RDB also exerts host-directed effects that complement its bacterium-directed actions. Effective resolution of *P. aeruginosa* infection requires efficient phagocytic clearance coupled with controlled inflammatory responses. Our findings indicate that RDB enhances macrophage-mediated bacterial uptake and clearance while restraining excessive production of key inflammatory mediators. This coordinated modulation suggests that RDB does not merely dampen bacterial virulence, but instead reshapes host – pathogen interactions toward a more balanced and protective immune state. The *in vivo* wound infection model integrates these mechanisms at the organismal level. The observed reduction in infection severity, limitation of systemic dissemination, and promotion of wound repair are consistent with simultaneous suppression of virulence signaling, disruption of biofilm stability, and optimization of host immune responses.

Several limitations warrant further investigation. Although computational and reporter-based analyses support multi-receptor QS antagonism, direct structural and biophysical characterization of RDB – receptor interactions will be necessary to confirm binding mechanisms. In addition, the molecular pathways underlying RDB-mediated immunomodulation remain to be defined, including whether these effects arise indirectly from altered bacterial signaling or from direct modulation of host pathways. Comprehensive pharmacokinetic and long-term safety studies will also be essential to evaluate translational potential.

In conclusion, RDB represents a marine-derived, dual bacterium- and host-directed anti-pathogenic agent that attenuates *P. aeruginosa* infection through multi-node QS suppression, biofilm regulatory interference, and balanced immune modulation, without inhibiting bacterial growth. By targeting both pathogen virulence networks and host inflammatory responses, RDB exemplifies a next-generation therapeutic strategy aimed at disarming rather than eradicating the pathogen, with potential advantages in limiting resistance development and improving outcomes in biofilm-associated infections.

## Supplementary Material

RDB revised Supplementary clean file.docx

## Data Availability

All original data and supplementary data are available in the figshare repository (https://doi.org/10.6084/m9.figshare.30490418) under a CC BY 4.0 license.

## Abbreviations

BHI: Brain heart infusion
c-di-GMP: Cyclic-di-GMP
eDNA: extracellular DNA
EPS: extracellular polymeric substance
ETA: exotoxin A
GFP: Green fluorescent protein
IL-1β: Interleukin-1 beta
IL-6: Interleukin-6
LB: Luria-Bertani
LPS: Lipopolysaccharides
M63-gly: M63minimal media supplemented with glycerol
PLC: Phospholipase
QS: Quorum sensing
TNF-α: Tumor necrosis factor α
TSB: Tryptic Soy Broth
