# Supplementary material for: A quorum sensing antagonist with dual bacterium- and host-directed anti-pathogenic activity against Pseudomonas aeruginosa
Source: Virulence. 2026 Aug 14;17(1):2712735. doi: 10.1080/21505594.2026.2712735 (PMC13479828; doi:10.1080/21505594.2026.2712735)
Supplement: Supplementary file 1 [file KVIR_A_2712735_SM3656.docx]

**A Quorum Sensing Antagonist with Dual Bacterium- and Host-Directed Anti-Pathogenic Activity against *Pseudomonas aeruginosa***

Dan-Dan Li^1-2#^, Huiyan Li^1#^, Wen-Xin Niu^1^, Huayue Li^4^, and Joon-Hee Lee^1,3*^

1. College of Pharmacy, Pusan National University, 46241, Busan, Republic of Korea; Institute for new drug

2. School of Chinese Materia Medica, Tianjin University of Traditional Chinese Medicine, Tianjin 301617, People's Republic of China

3. Research Institute for Drug Development, Pusan National University, Busan, 46241, South Korea

4. Key Laboratory of Marine Drugs, Ministry of Education, School of Medicine and Pharmacy, Ocean University of China, Qingdao 266003, China

**Corresponding Author:** Joon-Hee Lee: Laboratory of Microbiology, Department of Pharmacy, College of Pharmacy, Pusan National University. Research building 1009, Busandaehak-ro 63beon-gil 2, Jangjun-Dong, Geumjung-Gu, Busan 46241, South Korea. E-mail: joonhee@pusan.ac.kr

*#These authors contributed equally to this work.*

**Table S1. The Bacterial strains used in this study.**

| **Name** | **Description** |  | **References** |
| --- | --- | --- | --- |
| ***Pseudomonas aeruginosa*** | |  |  |
| PAO1 | Wild type *P. aeruginosa* |  | ^1^ |
| PAO1-GFP | PAO1 expressing chromosomally integrated GFP (green fluorescence protein) |  | ^2^ |
| PA14 | Clinical strain of *P. aeruginosa* |  | ^3^ |
| J16 | *P. aeruginosa* clinical isolate |  | ^4^ |
| MW1 | *lasI-, rhlI-* double mutant of PAO1 |  | ^5^ |
| ***Escherichia coli*** | |  |  |
| DH5α | *supE44*Δ*lacU*169(80*lacZ*ΔM15)*hsdR17 recA1gyrA96thi-1 relA1* |  | Lab.collection |
| ***Bacillus subtilis*** | |  |  |
| ATCC6051 | A wild type strain of *B. subtilis* |  | Lab.collection |
| ***Salmonella enterica*** | |  |  |
| SL1344 | A type of *S. enterica* serovar Typhimurium |  | Lab.collection |
| ***Staphylococcus aureus*** | |  |  |
| RN4220 | A wild type strain of *S. aureus* |  | Lab.collection |
| **Drug resistance strains** | |  |  |
| MDR1 | *P. aeruginosa* drug resistance strain (KNRRB 2200) |  | Lab.collection |
| MDR2 | *S. enterica* serovar Typhimurium (KNRRB 8173) |  | Lab.collection |
| MDR3 | *S. aureus* (KNRRB 3089) |  | Lab.collection |
| MDR5 | *Vibrio parahemolyticus* (KNRRB 7001) |  | Lab.collection |
| MDR6 | *Enterobacter cloacae* p 99 (KNRRB 0252) |  | Lab.collection |
| MDR7 | *E. coli* (KNRRB 1137) |  | Lab.collection |
| MDR8 | *Enterococcus faecium* (KNRRB 5207) |  | Lab.collection |
| MDR9 | *Enterococcus faecalis* (KNRRB 5210) |  | Lab.collection |

**Table S2. The plasmids used in this study.**

| **Plasmids** | **Description** | **References** |
| --- | --- | --- |
| pSC11 | *lasI*p-*lacZ* reporter in pQF50, ApR | ^6^ |
| pJL101 | *PA1897*p-*lacZ* reporter in pQF50, ApR | ^7^ |
| pJL501 | *rhlA*p-*lacZ* reporter in pQF50, ApR | ^8^ |
| pJL301 | *pqsA*p-*lacZ* reporter in pQF50, ApR | ^9^ |
| pJN105L | *lasR* orf in pJN105, GmR | ^7^ |
| pJN105Q | *qscR* orf in pJN105, GmR | ^7^ |
| pJN105P | *pqsR* orf in pJN105, GmR | ^9^ |
| pSKcdrA | *cdrA*p-*lacZ* reporter in pQF50, ApR | ^10^ |

ApR, ampicillin and carbenicillin resistance; GmR, gentamicin-resistance; orf, open reading frame.

**Table S3. The Interactions of RDB with LasR, QscR, RhlR and PqsR.**

| Interactions | | H-Bond | Hydrophobic bond |
| --- | --- | --- | --- |
| LasR  (4NG2) | Ligand (3OC12-HSL) | Trp60, Arg61, Asp73, Leu110 | Val76, Cys79, Leu125 |
|  | RDB | Thr115, Thr75 | Ala50, Leu40, Ala127, Val176, Phe101, Trp88, Ala105, Leu110, Trp60, Tyr64, Leu36 |
| PqsR  (6Q7U) | Ligand (HHQ) | Leu197, Ser196 | Ile236, Ile149, Leu208, Leu207 |
|  | RDB | Gln194, Pro210, Val211, Leu208 | Val211, Ile236, Ile263, Leu207, Leu208, Leu197, Pro129, Ala168, Ile149, Ala102, Pro238, Ala237 |
| QscR  (3SZT) | Ligand (3OC12-HSL) | Trp62, Ser38 | Met127, Pro117, Ile125 |
|  | RDB | Tyr58 | Tyr58, Tyr66, Trp90, Ile77, Val78, Asp75, Met127, |
| RhlR  (8B4A) | Ligand (C4-HSL) | Trp96, Thr121, Asp81, Trp68 | Val133, Ile84, Val160 |
|  | RDB | Ile84 | Val60, Tyr72, Leu69, Ala44, Ala111, Leu116, Trp96,Ala83 |

**Fig. S1.**

**Fig. S1.** The structures of 30 marine natural products used in this study.

**Fig. S2.**

**Fig. S2.** Inhibition of biofilm formation by RDB in PAO1, *E. coli, B. subtilis,* *S. aureus, S. enterica* (A) and *MDR* strains (B). The media used for each strain were as follows: PAO1, M63-Gly; *E. coli*, ¼-strength LB supplemented with 0.1% mannitol; *B. subtilis* and *S. aureus*, TSB supplemented with 0.5% glucose; *S. enterica*, LB supplemented with 2% glucose; MDR1, LB; MDR2–9, BHI.

**Fig. S3.**

**Fig. S3** Molecular dynamics simulation analysis of RDB binding to four QS regulators. For each target protein (LasR, PqsR, QscR, and RhlR), the left panel (A, D, G and J) shows the backbone RMSD trajectories of the apo protein and the corresponding RDB-bound complex over the 100 ns simulation, the middle panel (B, E, H and K) presents the residue-wise RMSF profiles, and the right panel (C, F, I and L) displays the variation in the number of intermolecular hydrogen bonds formed between RDB and the receptor during the simulation. The comparison demonstrates the influence of RDB binding on protein structural stability, local flexibility, and hydrogen-bond interactions.

## Reference

1.	Pearson JP, Pesci EC, Iglewski BH. Roles of Pseudomonas aeruginosa las and rhl quorum-sensing systems in control of elastase and rhamnolipid biosynthesis genes. J Bacteriol 1997; 179:5756–67.

2.	Winkler AM. Roles of Surrogate Membrane Lipids in Biofilm Formation of Pseudomonas aeruginosa. [Master's degree]. 2022, 6–11.

3.	Chung IY, Choi KB, Heo YJ, Cho YH. Effect of PEL exopolysaccharide on the wspF mutant phenotypes in Pseudomonas aeruginosa PA14. J Microbiol Biotechnol 2008; 18:1227–34.

4.	Kim SK, Li XH, Hwang HJ, Lee JH. Antibiofilm effect of biofilm-dispersing agents on clinical isolates of with various biofilm structures. J Microbiol 2018; 56:902–9.

5.	Whiteley M, Lee KM, Greenberg EP. Identification of genes controlled by quorum sensing in. P Natl Acad Sci USA 1999; 96:13904–9.

6.	Chugani SA, Whiteley M, Lee KM, D'Argenio D, Manoil C, Greenberg EP. QscR, a modulator of quorum-sensing signal synthesis and virulence in Pseudomonas aeruginosa. Proc Natl Acad Sci U S A 2001; 98:2752–7.

7.	Lee JH, Lequette Y, Greenberg EP. Activity of purified QscR, a Pseudomonas aeruginosa orphan quorum-sensing transcription factor. Mol Microbiol 2006; 59:602–9.

8.	Hwang HJ, Choi H, Hong S, Moon HR, Lee JH. Antipathogenic Compounds That Are Effective at Very Low Concentrations and Have Both Antibiofilm and Antivirulence Effects against Pseudomonas aeruginosa. Microbiol Spectr 2021; 9:e0024921.

9.	Choi Y, Park HY, Park SJ, Park SJ, Kim SK, Ha C, et al. Growth phase-differential quorum sensing regulation of anthranilate metabolism in Pseudomonas aeruginosa. Mol Cells 2011; 32:57–65.

10.	Kim SK, Park HY, Lee JH. Anthranilate deteriorates the structure of Pseudomonas aeruginosa biofilms and antagonizes the biofilm-enhancing indole effect. Appl Environ Microbiol 2015; 81:2328–38.
